# Advances in Universal CAR-T Cell Therapy

**DOI:** 10.3389/fimmu.2021.744823

**Published:** 2021-10-06

**Authors:** Haolong Lin, Jiali Cheng, Wei Mu, Jianfeng Zhou, Li Zhu

**Affiliations:** Department of Hematology, Tongji Hospital, Tongji Medical College, Huazhong University of Science and Technology, Wuhan, China

**Keywords:** cellular immunotherapy, chimeric antigen receptor T cell therapy, universal chimeric antigen receptor T cell therapy, gene editing, CRISPR/Cas9

## Abstract

Chimeric antigen receptor T (CAR-T) cell therapy achieved extraordinary achievements results in antitumor treatments, especially against hematological malignancies, where it leads to remarkable, long-term antineoplastic effects with higher target specificity. Nevertheless, some limitations persist in autologous CAR-T cell therapy, such as high costs, long manufacturing periods, and restricted cell sources. The development of a universal CAR-T (UCAR-T) cell therapy is an attractive breakthrough point that may overcome most of these drawbacks. Here, we review the progress and challenges in CAR-T cell therapy, especially focusing on comprehensive comparison in UCAR-T cell therapy to original CAR-T cell therapy. Furthermore, we summarize the developments and concerns about the safety and efficiency of UCAR-T cell therapy. Finally, we address other immune cells, which might be promising candidates as a complement for UCAR-T cells. Through a detailed overview, we describe the current landscape and explore the prospect of UCAR-T cell therapy.

## Introduction

With the vigorous development of cellular immunotherapy and the blowout of new clinical trials, various emerging cellular drugs have brought about a qualitative leap in the antineoplastic field. Chimeric antigen receptor T (CAR-T) therapy is the most rapid-developed and wide-applicated branch of anticancer cellular immunotherapy. This recent technology rapidly changed the landscape of hematological malignancies and already accounts for more than half of the cell therapies currently under development or in the market. As of March 2020, there were 1,483 anticancer cell therapies under research or on the market worldwide, with an increase of 46.7% compared with 1,011 in 2019. Among these, 858 are CAR-T cell therapies in 2020, a rise of more than 50% compared to the corresponding quarter last year (1).

**Figure 1:**
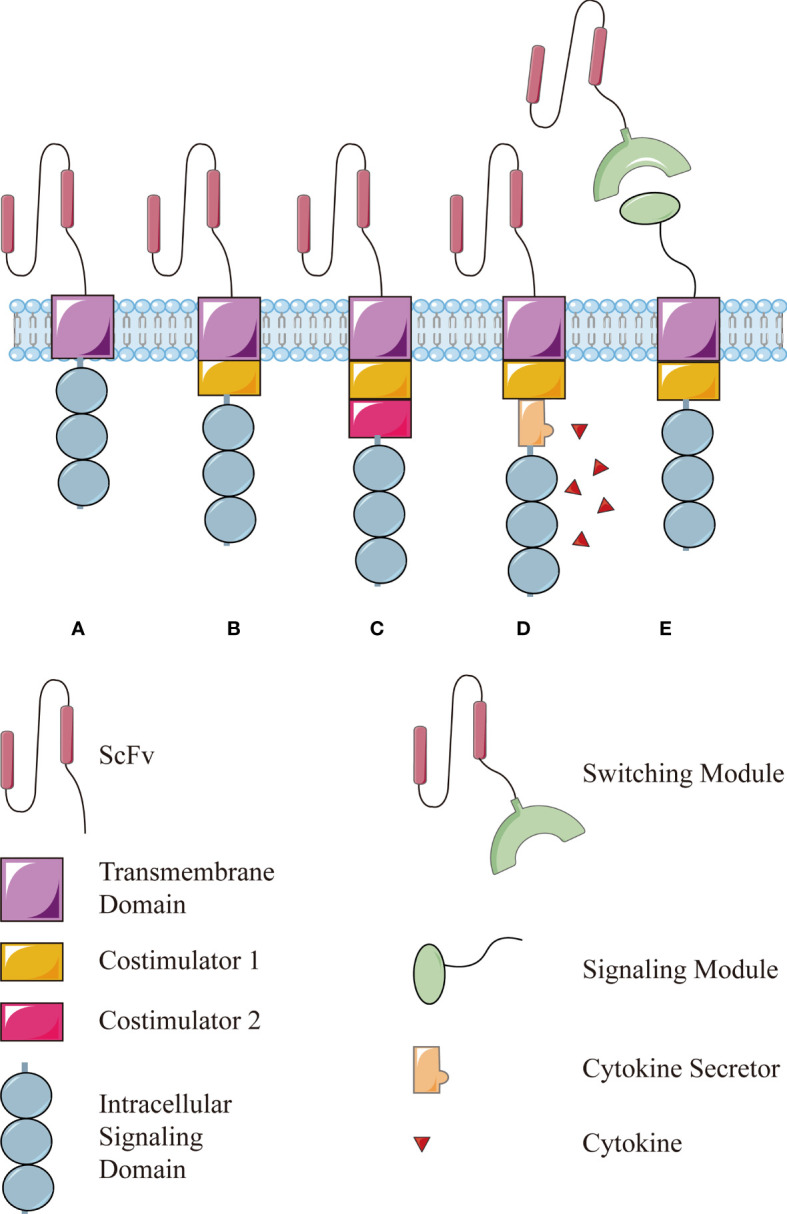
The structure of conventional CAR and modular CAR: **(A)** the first generation of CAR consists of an extracellular antigen-binding domain (usually the single chain variable fragment, scFv), a transmembrane domain, and an intracellular signaling domain of the CD3ζ chain. Then, a costimulator is added in the **(B)** second generation and more in the **(C)** third generation. **(D)** The fourth generation of CAR is modified further to secret a cytokine to enhance the function. **(E)** The modular CAR is split into two interactive parts, the signaling module on T cells and the switching module to recognize targets.

In a nutshell, this technology is based on T lymphocytes isolated from the circulation, which are then engineered to express chimeric antigen receptors (CARs), enabling modified T lymphocytes to recognize and respond to cancer cells independently of a major histocompatibility complex (MHC) engagement. After proliferation *in vitro*, these cells are reinfused into the patient to drive antitumor immune responses (2). The first generation of CAR used an extracellular antigen-binding domain (usually the single chain variable fragment of an antibody), a transmembrane domain, and an intracellular signaling domain of the CD3ζ chain (**Figure 1A**), simply driving a transient T-cell proliferation and limited cytokine secretion (3). Later, costimulatory molecules such as CD28 or 4-1BB were incorporated into CAR structure to promote CAR-T cells survival and functionality *in vivo*, leading to the second generation CAR (**Figure 1B**) (4) and then paired as the third generation of CAR structures (**Figure 1C**) (5). Recently CAR-T cells have been further modified to secreted cytokines such as interleukin (IL)-12, which enhances T-cell viability, recruits and activates other immune cells to enhance potency or safety (**Figure 1D**) (6–8).

The second-generation CAR-T cell is the most effective and widely used. Five CAR-T cell products, namely, Kymriah (tisagenlecleucel, tisa-cel), Yescarta (axicabtagene ciloleucel, Axi-Cel), Tecartus (brexucabtagene autoleucel, KTE-X19), Breyanzi (lisocabtagene maraleucel, liso-cel), and Abecma (idecabtagene vicleucel, Ide-cel), have been approved by the Food and Drug Administration (FDA) for clinical treatment in relapsed or refractory acute B lymphoblastic leukemia, B lymphoid malignancies, and multiple myeloma, respectively. In China, Yescarta was the first approved CAR-T cell product released on the market on June 22, 2021. Relma-Cel, another anti-CD19 CAR-T cell product, is under premarket review as well. In the most recent reports, objective remission rates of Kymriah and Yescarta in the treatment of relapsed or refractory B non-Hodgkin’s lymphoma have reached 52% and 82%, respectively (9, 10).

Nonetheless, some limitations hinder the dissemination and development of CAR-T cell therapy. First, many factors may lead to the failure of CAR-T cell therapy, including the intrinsic factors (such as poor CAR-T cell expansion or short persistence) and extrinsic factors (tumor cells with target deletions or mutations and tumor inhibitory microenvironment) (11). Second, the safety concerns still need to be addressed. CAR-T cells drive tumor clearance but can also lead to potentially lethal toxicity, including cytokine release syndrome (CRS) and neurotoxicity caused by CAR-T cells overactivation, excessive cytokine release, and “on-target/off-tumor effect” due to low specificity of antigen expression (12, 13). In addition, the high cost and the labor-intensive manufacturing process of CAR-T cells still hamper the popularization of CAR-T cell therapy. A one-time infusion of Kymriah costs $475,000, and the total cost for Kymriah or Yescarta treatment is nearly 1 million dollars per patient (14). Furthermore, the current production cycle takes about 2 weeks, during which highly proliferative malignancies continue to progress (15). Moreover, cancer patients frequently suffer from congenital immunodeficiency or lymphocytopenia after repeated chemotherapies, resulting in suboptimal T cells inadequate for CAR-T cell manufacturing. Rarely, but worst of all, if leukemic blasts contaminate isolated lymphocytes and are inadvertently loaded with CAR, they can mask the targets and escape from CAR-T cells. Until now, there was only one reported case where leukemic B cell was unintentionally modified by CD19-CAR, conferring resistance to CD19 CAR-T cells and leading to lethal complications related to progressive leukemia ultimately (16). All of these pitfalls cast a shadow over the development of CAR-T cell therapy (17).

Currently, universal CAR-T (UCAR-T) cell therapy is in the spotlight and expected to break the plight. All existing CAR-T cell products on the market or under testing are autologous (made with same patient-derived T lymphocytes) to avoid severe alloimmune rejection due to a mismatch of MHC between the donor and the recipient. Alternatively, UCAR-T cells would consist of allogeneic CAR-T cells that are taken from healthy donors. Despite sharing the same killing mechanism, UCAR-T cells have distinct manufacturing processes, cost, safety considerations, and applicability (**Table 1**) (18). When customized CAR-T cell therapy can evolve into a universal therapy, many of the flaws that impede CAR-T cell dissemination can be readily addressed. Finally, large-scale production procedures and batch manufacturing could greatly increase the quality and accessibility of CAR-T cell products.

**Table 1:** The comparison of autologous and allogeneic CAR-T cell therapy.

	Autologous CAR-T cell therapy	Universal CAR-T cell therapy
**Consistency**		
Killing mechanism	MHC-independent	
Gene editing to avoid fratricide	Carried out if needed	
Manufacturing process	T lymphocytes are isolated and transduced with a specific CAR by viral vector, then refused to the patient after amplification	
**Difference**		
Cell source	Patients themselves	Healthy donors
Activation of the immune system in patients	Hardly	Possible
Manufacturing Line	Customized	Batched
Additional Gene Editing to avoid GVHD and rejection	Unnecessary	Necessary
Cost	High	Much lower
Immediate availability	No	Yes
Application in T-cell malignancies	Restricted	Promising
Main risks	CRS;CRES	CRS;CRES;GVHD
Limitations	Suboptimal quantity and quality of T cells in patients	Lower amplification and shorter persistence *in vivo*

CAR, chimeric antigen receptor; CRS, cytokine release syndrome; CRES, CAR-T cell-associated encephalopathy syndrome; GVHD, graft versus host disease.

## The Evolution of UCAR-T Cell Therapy

The concept of allogeneic CAR-T cell therapy has persisted for a long time. In relapsed patients, successfully treated by allogeneic hematopoietic stem cell transplantation (allo-HSCT), CAR-T cells can be produced from the transplant donors or recipients, but the efficacy and safety of each are still uncertain. In an early study (NCT01087294), 10 persistent patients with B-cell lymphoma or leukemia after allo-HCST and standard donor lymphocyte infusions received transplant donors-derived allogeneic CAR-T cells without lymphodepletion. Three of them showed tumor regression, but none of these patients showed graft versus host disease (GVHD) (19). In another study with longer follow-up, 8 [6 complete responses (CRs) and 2 partial response (PRs)] of 20 patients entered remission, with none developing new-onset acute GVHD and only 2 with mild chronic GVHD after CAR-T cells refusion (20). In contrast, a similar study (NCT01864889) reported grade 2–3 GVHD in two patients 4 weeks after donor-derived CAR-T cells infusions (21). Recently, a retrospective study compared 14 patients receiving allogeneic CAR-T cells (3 donor-derived and 11 recipient-derived) after HSCT with 17 patients receiving autologous CAR-T cells (22). These showed no significant difference between autologous CAR-T cells and recipient-derived allogeneic CAR-T cell therapy on CR rate and long-term survival, but the latter with significantly lower proliferation and decreased cytokine release reaction. In this study, only two recipient-derived (18.2%) and 1 donor-derived (33.3%) allogeneic CAR-T cells caused acute GVHD (22).

These inconsistent results of GVHD may be explained by chronic hyperactivation, accelerated exhaustion, and activation-induced cell death (AICD), resulting from double stimulation from T-cell receptor (TCR) and CAR on allogeneic CAR-T cells. In a donor-derived allogeneic CAR-T cell mouse model, Arnab Ghosh et al. demonstrated that allogeneic CAR-T cells could be activated by CAR and TCR, respectively; however, activation of one receptor could restrain the function of the other. Hence, GVHD was alleviated when CD19-positive cells activated allogeneic CAR-T cells (via anti-CD19 CAR) before TCR-engagement by alloantigen. Therefore, they recommended that allogeneic CAR-T cells should be transfused only after B lymphocytes recovering from transplantation (23). A contradictory report that only CD19-positive leukemia could drive allogeneic activation of CAR-T cells and mediate acute GVHD. When activated by tumor cells, allogeneic CAR-T cells showed more severe rejection to the alloantigen (24). This discrepancy may be related to the degree of activation of UCAR-T cells. When the stimulation of CAR by target antigen is moderate, allogeneic CAR-T cell is activated but not excessively, driving an effective response to alloantigen. But when CD19 stimulation is overly strong, CAR-T cells become exhausted and unresponsive to allogeneic antigens. This suggests a delicate relationship between CAR and TCR in constant competition and collaboration. Given the complexity of dual signal controlled by TCR and CAR, the elimination of GVHD by disrupting TCR has become a strategy adopted by most allogeneic CAR T-cell researchers.

This strategy of transplant bridging to a recipient or donor-derived CAR-T cell therapy is stranded in one-to-one correspondence, far from the envisaged one-to-many universalization. With the accumulation of experience in allogeneic CAR-T cells, the production of “off-the-shelf” CAR-T cells from third-party healthy donors has been put on the agenda. At the American Society of Hematology (ASH) meeting in December 2017, Cellectis announced the preliminary results of two clinical trials of UCART19, and since then, universal CAR-T cell therapy has officially come into the public sight.

## Recent Developments in UCAR-T Cell Therapy

### Targets of UCAR-T Cell Therapy

There have been more than hundreds of preclinical and clinical trials of allogeneic CAR-T cell therapy worldwide (18, 25). The majority of these are applied to hematological malignancies, where the most popular target is CD19, and other classic targets, including CD20, CD22, and BCMA. New developing targets such as CD70, CD7, and CD5 are also included (18, 26). NKG2DL, GD2, and mesothelin for solid tumors are also emerging (**Table 2**) (18, 29, 30).

**Table 2:** Summary of targets involved and strategies to improve the efficiency in UCAR-T cell therapy.

Target	UCAR-T product	Improving strategies	Editing tools	Development phase	Reference/NCT number
CD 19	UCART019	TRAC and B2M KO	CRISPR/Cas9	Phase I/II	NCT03166878
	CTX110	TRAC and B2M KO	CRISPR/Cas9	Phase I	NCT04035434
	/	TRAC, B2M and PD-1 KO	CRISPR/Cas9	Preclinical	(27)
	UCART19/ALLO-501	TRAC KO with or without CD52 KO	TALEN	Phase I	NCT02735083; NCT02808442; NCT02746952;
	FT819	TRAC KO and iPSC-derived T cells	CRISPR/Cas9	Phase I	NCT04629729;
BCMA	CTX120	TRAC and B2M KO	CRISPR/Cas9	Phase I	NCT04244656
CD123	UCART123	TRAC KO	TALEN	Phase I	NCT03190278; NCT03203369
CD22	UCART-22	TRAC and CD52 KO	TALEN	Phase I	NCT04150497
CS1	UCARTCS1A	TRAC and CS1 KO	TALEN	Phase I	NCT04142619
CD19/CD20; CD19/CD22	Universal dual specificity CAR-T cells	TRAC KO	CRISPR/Cas9	Phase I/II	NCT03398967
CD5	CT125A	TRAC and CD5 KO	CRISPR/Cas9	Phase I	NCT04767308
CD7	GC027	TRAC and CD7 KO	CRISPR/Cas9	Phase I	(28)
	UCART7	TRAC and CD7 KO	CRISPR/Cas9	Preclinical	(26)
CD70	CTX130	TRAC and B2M KO	CRISPR/Cas9	Phase I	NCT04438083; NCT04502446
Mesothelin	/	TRAC and PD1 KO	CRISPR/Cas9	Phase I	NCT03545815
NKG2D	CYAD-101	TIM peptide of CD3ζ	Retroviral vector	Phase I	NCT03692429
NKG2DL	CTM-N2D	γδ T Cells	/	Phase I	NCT04107142
GD2	/	EBV-CTLs	/	Phase I	NCT00085930

TRAC, T-cell receptor alpha constant chain; B2M, beta-2-microglobulin; PD-1, programmed cell death protein 1; CRISPR/Cas9, clustered regularly interspaced short palindromic repeats/Cas9; TALEN, transcription activator-like effector nuclease; iPSC, induced pluripotent stem cell; BCMA, B-cell maturation protein; TIM peptide, TIM peptide TRAC-inhibitory molecule peptide; EBV-CTLs, Epstein–Barr virus-specific cytotoxic T lymphocytes; KO, knockout.

Allogene Therapeutics was the forerunner in this UCAR-T field with UCART19. Two multicenter phase I clinical trials (NCT02808442 and NCT02746952) aiming to investigate the safety, feasibility, and antileukemic activity of UCART19 in children and adults with relapsed or refractory B-cell acute lymphoblastic leukemia have been conducted. Seven children and 14 adults were enrolled, of which 14 (14/21, 67%) patients had a complete response or complete response with incomplete hematological recovery 28 days after infusion. CRS (19/21, 91%) was the most common adverse side effect, of which 3 (3/21, 14%) were grade 3–4. Other adverse events included mild neurotoxicity (8/21, 38%), grade 4 prolonged cytopenia (6/21, 32%), and grade 1 acute skin GVHD (2/21,10%). Two treatment-related deaths were reported as a result of neutropenic sepsis and pulmonary hemorrhage, respectively (31). Two infants mentioned above acquired molecular remission and bridged to allogeneic HSCT successfully (32). UCART19 is undoubtedly a remarkable step forward in the field of UCAR-T cells, and it offers an opportunity for patients with rapidly progressive diseases who cannot access autologous CAR-T cell therapy.

In addition to CD19, targets of UCAR-T cell products being developed by Allogene Therapeutics include CD123 (NCT03190278, NCT03203369), CD22 (NCT04150497), BCMA (NCT04093596), and CS1 (NCT04142619). Unlike the smooth progress of CD19, the CD123 program has been full of twists and turns. In November 2017, after one death in the clinical trial (NCT04106076), it was announced that UCART123 would continue two phase I clinical trials for acute myeloid leukemia (NCT03190278) and blastic plasmacytoid dendritic cell neoplasm (NCT03203369) subject to agreed clinical regimens with FDA. The detailed results are still unknown.

Most research targeted one specific marker, but UCAR-T cell allows for a CD19/CD20 and CD19/CD22 (NCT03398967) multitarget approach. Recently, Yongxian Hu et al. reported CTA101, a universal CD19/CD22 dual-targeted CAR-T cell that disrupted T-cell receptor alpha chain (TRAC) and CD52 by clustered regularly interspaced short palindromic repeats/Cas9 (CRISPR/Cas9). This exhibited a CR rate of 83.3% (5/6) without dose-limiting toxicity, GVHD, neurotoxicity, or adverse events related to genome editing (33).

Currently, there are just a few registered UCAR-T cells clinical trials for solid tumors, such as allogeneic NKG2DL-targeting CAR-T cells (NCT04107142) for relapsed or refractory colorectal cancer, breast cancer, and sarcoma. Additionally, allogeneic disialoganglioside 2 (GD2)-targeting CAR-T cells are under test for relapsed or refractory neuroblastoma (NCT01460901) and allogeneic CD70 targeting CAR-T cells for relapsed or refractory renal cell carcinoma (NCT02830724). The latter has been suspended. Based on these clinical trials, it is likely that UCAR-T cell therapy will be first used for hematological malignancies, while for solid tumors, the UCAR-T cell study is still in its infancy with broad prospects for the future.

### Gene Editing in UCAR-T Cell Therapy

**Figure 2:**
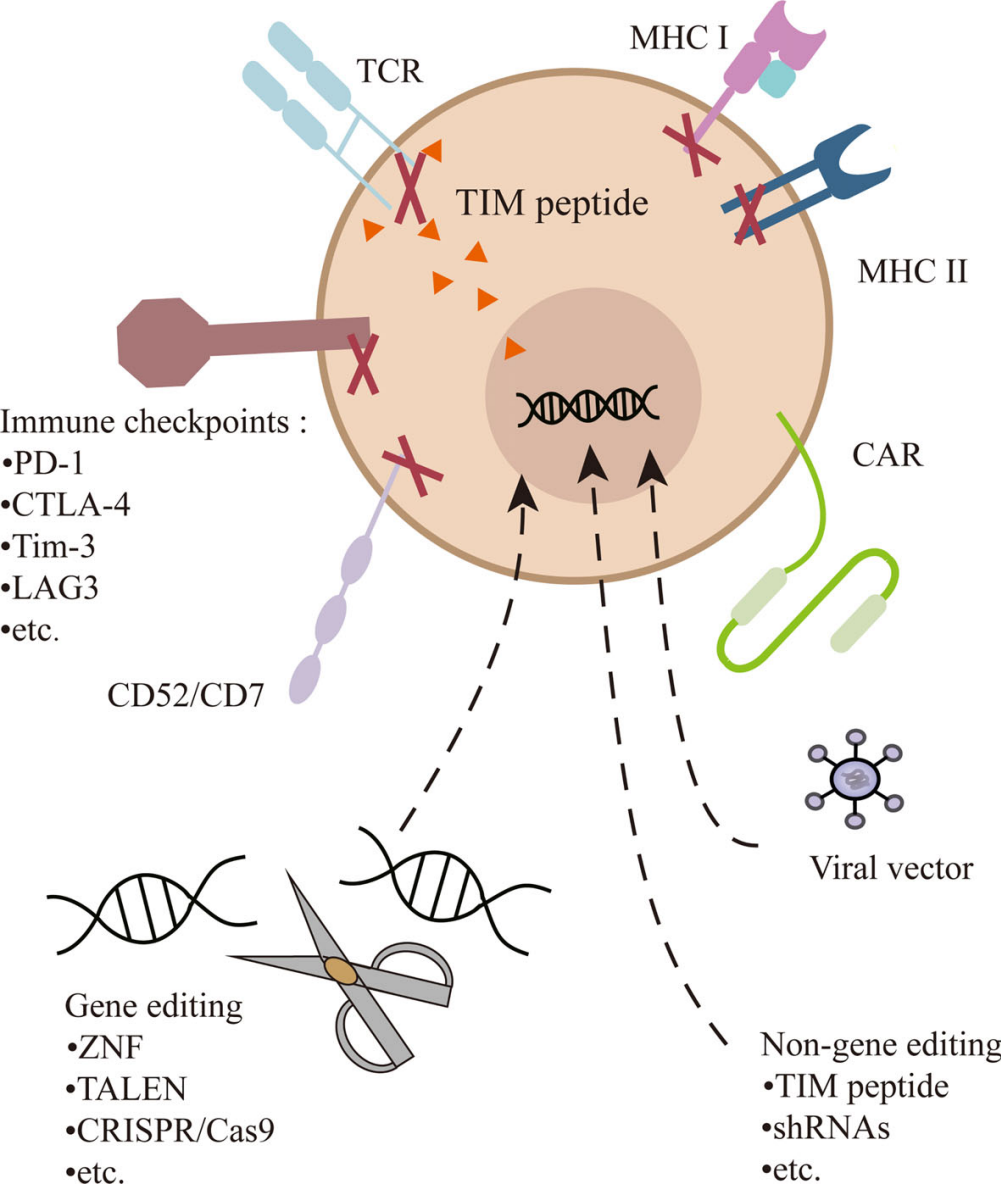
Multiple gene or non-gene editing on UCAR-T cells. In addition to transducing a CAR into T cells, the TCR can be knocked out or inhibited to prevent GVHD. Genetic ablation of MHC-I and/or MHC-II diminish immunogenicity. Destruction of CD52 can make cells resistant to alemtuzumab. CD7 is edited to prevent the fratricide in CD7 UCAR-T cells. In addition, inhibitory checkpoints (e.g., PD-1) can be knocked out to enhance the function of cells.

The CAR-T cell is commonly transduction with viral vectors, mostly lentiviral vectors, which have an advantageous transfection efficiency and stable expression. However, random genome integration raises the risk of insertion mutation and disruption of functional genes (34). Therefore, the development of UCAR-T depends on the progress of gene-editing technology. A variety of gene-editing methods have been applied to improve transduction efficiency, diminish GVHD, and enhance persistence. Zinc-finger nucleases (ZFN) (35), transcription activator-like nucleases (TALENs) (36), and CRISPR/Cas9 (25, 33) can all achieve positional editing in the genome and have been employed in UCAR-T cell therapy. TALENs is most adopted by Allogene Therapeutics, and CRISPR/Cas9 offers even greater flexibility, maneuverability, and relative accuracy, opening the possibility of multiple gene editing (**Figure 2**). Currently, it has been employed in several clinical trials, including UCART019 targeting CD19 (NCT03166878), CTX130 targeting CD70 (NCT04502446, NCT04438083), CTX120 targeting BCMA (NCT04244656), and CT125a targeting CD5 (NCT04767308). For the expression of CAR in check, CD19-specific CAR is knocked into the TRAC locus of T cells, by which its expression is enhanced and unified under the control of the TCR promoter (37, 38). In UCART7 targeting CD7 for T-cell malignancies, TRAC and CD7 are simultaneously knocked down, the former for preventing GVHD and the latter for preventing fratricide of the very effector cells (26).

On the other hand, non-gene editing universal CAR-T therapy has also achieved initial results. Celyad has conducted several clinical trials with the CYAD-101, a non-gene editing natural killer group 2D (NKG2D)-based UCAR-T cell product, in both solid and hematological tumor types. It tampered with or eliminated TCR signals and reduced GVHD by expressing a TRAC-inhibitory molecule (TIM) peptide. The preliminary results of the phase I trial showed no evidence of CYAD-101 causing GVHD in the treatment of metastatic colorectal cancer. Using the short hairpin RNA (shRNA) platform of Horizon, Celyad has developed the next generation non-gene edited allogeneic CYAD-200 series of CAR-T candidates.

### Modularization and Logic Gating

Gene editing transforms T cells from third-party healthy donors to a stable and universal cell resource, while the development of CAR structure makes it possible to design a CAR for multiple targets at the same time, the combination of which enables the idea of an upgraded UCAR-T (5).

In 2012, Urbanska et al. proposed a modular CAR design composed of extracellular-modified avidin linked to an intracellular T-cell signaling domain. These modified T cells recognized and bound exclusively to cancer cells pretargeted with specific biotinylated junction molecules, such as biotinylated antibodies (39). Despite the high immunogenicity in humans, this idea opened the door to the modularization of CAR structure (**Figure 1E**). The CAR is split into two parts: (i) the signaling module on T cells, consisting of the extracellular domain that specifically binds to the switching module and the intracellular domain that transmits the activation signals; (ii) the switching module, usually a bispecific antibody or small molecule recognized by the signaling module on T cells and binding to the targets on cancer cells. This split, universal, and programmable (SUPRA) CAR system currently adopts a variety of recognition modes including neo-epitopes, SpyTag, biotin, and fluorescein isothiocyanate (FITC) and leucine zippers (40). Clinical trials of SUPRA CAR have been carried out for CD19/CD20 (NCT02776813) and CD123 (NCT04230265). Other targets under development include CD33, prostate stem cell antigen (PSCA), prostate-specific membrane antigen (PSMA), GD2, epidermal growth factor receptor (EGFR), cell-surface-associated mucin 1 (MUC1), and sialyl-Tn (STn) (29). What is more, the CD123-specific targeting module has been further deimmunized to mitigate the potential immunogenicity, which proved its good tolerance and targeting effect in the human body (41).

This flexible CAR structure changes the original rigid structure of CAR to improve security and feasibility. As a bridge between CAR-T cells and tumor cells, the dosage of the switching module can be titrated because it conforms to general pharmacokinetics, and its affinity to target antigens can be regulated by fine-tuning the structure to take control of CAR-T cell activation. Besides, CAR-T cells are held back by blocking agents, which competitively inhibit switching modules when necessary.

Recently, a photo-switchable CAR-T cell with dose-dependent and rapidly terminated cytotoxicity has appeared. Switching modules carrying dual folate and FITC tethered by an ortho-nitrobenzyl ester photocleavable linker (folate-O-FITC), CAR-T cells are turned off under the light of 365 nm, in which switching modules were snipped and activated again by resupplementation with the mediator (42). These make it possible to accurately predict and control the activation of CAR-T cells and the release of cytokines. When various switching modules are injected simultaneously, multitarget CAR-T cell therapy can be easily achieved without altering cells, which is promising in preventing targets mutation (43, 44).

**Figure 3:**
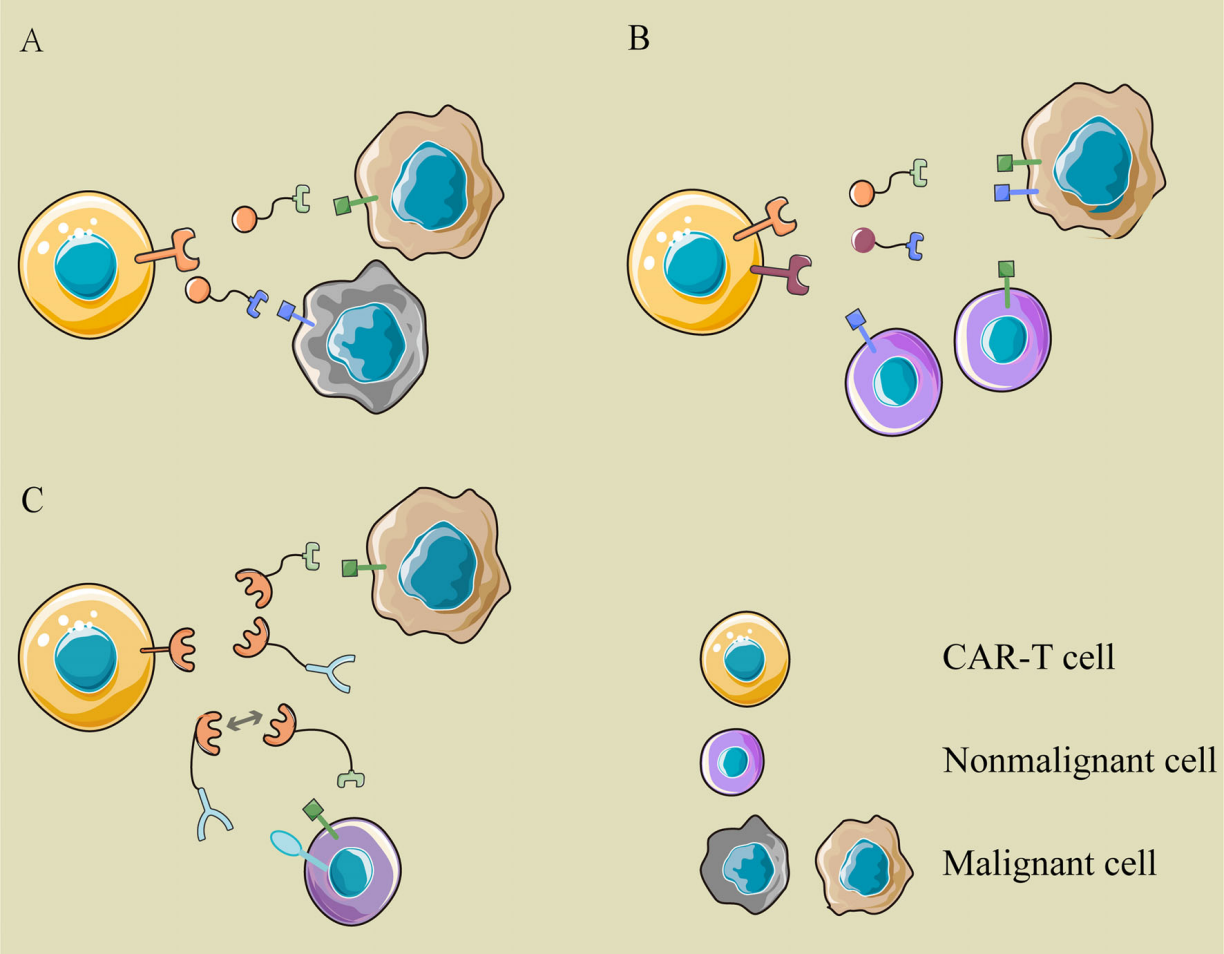
The logic gatings in modular CAR. **(A)** OR logic: the modular CAR-T cell can eliminate different cancer cells with various switching modules, which are recognized by the same CAR-T cell but target different antigens on cancer cells. **(B)** AND logic: the antigen-binding domain and costimulator are separated into two CARs targeting different antigens and cotransduced into T cells. Only when tumor cells express two antigens simultaneously can they be recognized and attacked by these CAR-T cells. **(C)** NOT logic: a tumor-associated antigen is expressed on cancer cells and normal cells simultaneously, while another antigen is expressed on normal cells only. The two modules binding to them are complementary in the site recognized by the signaling module. The extra target works as a safety label to prevent the “on-target, off-tumor” toxicity of CAR-T cells.

What is even more exhilarating is the logical control of CAR-T cells through multiple switching modules. Existing bispecific CAR T-cell therapy adopts “OR” logic, in which CAR-T cells are activated if the tumor cells express a single target (**Figure 3A**). The modular CAR design can function “AND” and “NOT” gate to promote selective tumor eradication without on-target, off-tumor toxicity (45, 46). For “AND” gating strategies, the antigen-binding domain and costimulatory domain can be separated into two CARs targeting different antigens and cotransduced into T cells (**Figure 3B**). Aiming at two tumor-associated antigens, PSMA and PSCA, Christopher Kloss et al. constructed such an “AND” logic bispecific CAR-T cell, which destroyed tumors that expressed both PSMA and PSCA but did not work on tumors expressing either alone (45). Similarly, the modular CAR system can perform the “NOT” logic to increase tumor specificity through combinatorial antigens (**Figure 3C**). For instance, a SUPRA CAR system targeted cells expressing Her2 only and spared cells expressing Her2 and Axl both. In this design, both Her2- and Axl-positive cells are bound to two switching modules, α-Her2-EE zipFv and α-Axl-SYN2 zipFv, simultaneously. Then, these two modules recognized and combined with each other by zipFv, so they failed to activate CAR-T cells (47). This suggests that when tumor-associated antigens are also expressed on normal cells, an additional target can be combined as a “safety label” to further distinguish normal cells from tumor cells.

When modular CAR is adopted in UCAR-T cells, the ultimate goal of treating different cancers with cells of stable source and CAR of identical structure makes solid progress.

## Challenges in UCAR-T Cell Therapy

### Safety of UCAR-T Cell Therapy

Allogeneic cells and complex gene editing make people more cautious about UCAR-T cell therapy. The existing risks in autologous CAR-T cell therapy, such as CRS and neurotoxicity, cannot be ruled out in UCAR-T cell therapy. However, the GVHD is the first and foremost challenge that hinders the realization of this therapy. It is logical to knock out the TCR on cells and then enrich the TCR-negative UCAR-T cells for reinfusion. The improvements in gene editing make this technically achievable (48). However, gene editing is not necessarily a complete gospel. Complex genetic manipulation increases the risk of unexpected gene mutations (49). Safe and efficient gene manipulation is still being explored. What is more, higher-dose lymphodepletion chemotherapy in UCAR-T cell therapy is accompanied by the increased risk of opportunistic infection. All of these can be fatal for patients.

### Efficiency of UCAR-T Cell Therapy

CAR T cells should undergo a process of proliferation and then persist *in vivo*. Cytokinetics revealed the comparable early expansion but shorter persistence in allogeneic CAR T-cells than autologous CAR-T cells and failure to generate a memory pool (24). In related clinical trials, the failure of UCAR-T cells to expand and maintain sufficient levels in patients remains a major concern. This can be solved by alleviating the host rejection or reducing the immunogenicity of infused cells (**Table 2**).

Increasing clinical practice shows that the lymphodepletion chemotherapy before cell infusion creates a favorable environment for the expansion of CAR-T cells *in vivo*. The commonly used conditioning regimens are fludarabine combined with cyclophosphamide, but more exhaustive lymphodepletion has been applied in UCAR-T cell therapy. In the landmark clinical trial of UCART19 (NCT03939026), T cells were engineered to knock out genes encoding TCRA and CD52, to disrupt the structure of TCR and acquire resistance to anti-CD52 monoclonal antibody alemtuzumab, since CD52 is both positive in T and natural killer (NK) cells, which eliminate the allogeneic CAR-T cells in recipients. The addition of alemtuzumab can further suppress the allogeneic immune rejection in hosts and extend the therapeutic window for the amplification of UCAR-T cells. It was clear that all patients with CR were pretreated with fludarabine, cyclophosphamide, and alemtuzumab (14/17, 82.4%), but none of the four patients without alemtuzumab showed UCART19 expansion or antileukemic activity. The finding illustrated the absolute necessity of a powerful and thorough lymphodepletion for UCAR-T cells amplification (31). Similar gene-modifying and pretreatments were found in CTA101, a CRISPR/Cas9-engineered universal CD19/CD22 dual-targeted CAR-T cells (33).

Except in combination with CD52 knocking out, UCAR-T cells resistant to traditional chemotherapeutics have also been designed. Purine and pyrimidine nucleoside analogues, as common chemotherapeutic agents, such as clofarabine, fludarabine, and cytarabine, take effect only after being metabolized by deoxycytidine kinase (dCK). TCR-negative and chemotherapeutics-resistant UCAR-T cells were obtained by employing TALEN to block the expression of TRAC and dCK, which made it possible to lymphodeplete repeatedly whenever lymphocytes recover without impacting UCAR-T cells unintentionally. Besides, lymphocytes of the recipient might restore by breaking off lymphodepletion and remove overkilling UCAR T-cells to prevent severe toxicity (50).

Like CD52, CD7 is a transmembrane glycoprotein with expression on T cells and NK cells, and it is also a target of great concern in T-cell tumors. In CD7 UCAR-T targeting T-cell malignancies, TCR and CD7 are also knocked out to avoid GVHD and fratricide between effector cells, respectively. In addition to malignant T cells, CD7 UCART can recognize normal T and NK cells as well, resulting in more lasting lymphodepletion. Mathew et al. reported that this UCAR-T cell kept robust antileukemia effect in cell lines and primary T-cell acute lymphoblastic leukemia (T-ALL) blasts *in vitro* and in NSG mice, and no fratricide or GVHD was found (26). Recently, an open-label and single-arm clinical trial of GC027, a CD7 UCAR-T of TCR and CD7 edited by CRISPR, was published in two patients with refractory/relapsed T-ALL after potent lymphodepletion (fludarabine, cyclophosphamide, and prednisone) and a single infusion of GC027. Both patients achieved CR with negative minimal residual disease, and one remained ongoing remission at cutoff (28).

Thoroughly, lymphodepletion is accompanied by serious T-cell aplasia. Different from B-cell aplasia, which can be compensated by periodic infusions of intravenous immunoglobulins, the persistent deficiency of T and NK cells is life threatening. Ideally, one would suppress immunological rejection but retain part of the immune protection. One of the characteristics of alloreactive T and NK cells is the upregulation of 4-1BB on their surface (51, 52). Feiyan Mo et al. engineered an alloimmune defense receptor that identified and attacked 4-1BB upregulated lymphocytes and coexpressed it in allogeneic CAR-T cells. These therapeutic cells could eliminate alloimmune lymphocytes and tumor cells simultaneously but leave resting T and NK cells alone. Later, they found that these CAR-T cells produced sustained tumor eradication without being rejected in mice (53). Although it is still in the preclinical stage, this study can drastically shift the paradigm of prolonging the persistence in UCAR-T cell therapy and broaden its applicability.

Apart from suppressing the immune system in hosts, reducing the immunogenicity of UCAR-T cells is another approach to enhance its persistence. MHC is the major antigen system driving graft rejection. MHC-I is expressed on the surface of almost all living cells in human; therefore, inhibiting the expression of MHC-I can evade the attack of alloreactive T cells in recipients. CRISPR Therapeutics has been taking such an approach, including CT110 targeting CD19, CTX120 targeting BCMA, and CTX130 targeting CD70. Endogenous TCRA and β-2 microglobulin(B2M) genes are disrupted simultaneously by applying CRISPR RNA electroporation to manufacture UCAR-T cells, which are both TCR and MHC-I negative, aiming to evade rejection and deliver antileukemic effects without GVHD, but the results of these studies are still unpublished. Another upgrade study was to generate gene-disrupted allogeneic CAR-T cells deficient in TCR, MHC-I, and PD-1, which demonstrated reduced alloreactivity and enhanced antitumor activity *in vivo* without causing GVHD (27).

Although UCAR-T cells are exempt from alloreactive T cells by B2M knocking out, another militant, the NK cells, are activated in the absence of MHC-I on UCAR-T cells and evolve into the main force in the elimination of UCAR-T cells. Several strategies have been tried to inhibit or clear reactive NK cells in recipients, but it is not easy to adopt a broad strategy to suppress all NK cells for the heterogeneity of NK cells. Upregulation of human leukocyte antigen (HLA)-E on UCAR-T cells, for example, showed inhibition of a subset of NK cells by binding to NKG2A/B receptors while stimulating another group of NK cells by activating the NKG2C (54), but more studies are needed to achieve the inhibition of activated NK cells.

MHC-II molecule is the subordinate factor to mediate alloimmune rejection by CD4^+^T cells, and its expression is regulated by regulatory factor X ankyrin repeat-containing protein (RFXANK) and class II MHC transactivator (CIITA) (55, 56). Allogeneic anti-CD19 CAR T cells with B2M, CIITA, and TRAC triple knocking out showed better persistence when cultured with allogenic peripheral blood mononuclear cells (PBMCs) than TRAC and B2M double knocking-out CAR-T cells, without altering the function of T cells (57). Similar engineering in iPSC was conducted to disrupt B2M, CIITA, and CD155 (encoding an activating ligand of NK cells) and transduce HLA-E, serving as a source of CAR-T cells. These hypoimmunogenic CAR-T cells largely escaped rejection by CD8^+^T cells, CD4^+^T cells, and NK cells, maintaining antitumor cytotoxicity (58).

Multiple gene editing strategies reduce rejection of UCAR-T cells *in vivo*. On the other hand, increasing accessibility and further ablation of immunogenicity in UCAR-T cells allows for multiple reinjections, making CAR-T cell therapy more like conventional drugs, in which efficacy and side effects can be easily controlled by repeated and transient infusions of cells.

## Alternative Universal Cell Therapies

At present, most CAR-T cells are derived from T cells in PBMCs. However, other types of cells may have unique advantages in the process of universalizing the cell therapy, as a supplement or substitutions of UCAR-T cell therapy (59).

### Other Subpopulations of T Cells

Certain subsets of T cells with unique superiority in mitigating GVHD are also promising candidates for producing UCAR-T cells. Based on the peptide chain structure of TCR, T cells are divided into αβT cells consisting of α and β chains and γδT cells with γ and δ chains. Despite in lower frequencies, γδT cells play an important role in the innate immune response and anti-infective or antitumor reaction independent of the MHC or antigen-presenting cells (APCs) (60, 61). In antitumor immunity, γδT cells recognize and eliminate tumor cells independent of TCR, which responds to a specific tumor-associated antigen (TAA) (62, 63). These characteristics endow γδT cells with inherent advantages in cellular immunotherapy in solid tumors that lack specific TAAs. Anna Capsomidis et al. reported that GD2-CAR γδT could amplify *in vitro* retaining antigen-presenting function and the GD2-targeting ability (64). A registered clinical research (NCT04107142) based on allogeneic NKG2DL-targeting CAR γδT cells against multiple solid tumors, including colorectal cancer, breast cancer, sarcoma, nasopharyngeal cancer, prostate cancer, and gastric cancer, is still in phase I.

Invariant natural killer T (iNKT) cells are another cell subpopulation that share characteristics of NK and T cells, and they have striking intrinsic antitumor activity for their endogenous TCR, which restrictedly recognizes foreign lipid antigens in the context of CD1d (65, 66). It has been reported that adoptive transferred iNKT cells are able to exert graft versus leukemia (GVL) but suppress GVHD after HSCT in leukemia patients (67). Previous studies have shown that iNKT cells engineered with CAR have equivalent or better cytotoxicity with a better safety profile than conventional CAR-T cells in solid tumors (66, 68, 69). A clinical study of allogeneic CAR19-iNKT cells for hematological malignancy (NCT03774654) is ongoing (70).

In addition, regulatory T cells expressing chimeric antigen receptors (CAR-Tregs) have been tried in autoimmune diseases to induce immune tolerance after organ transplantation (71, 72).

### Natural Killer Cells

Compared with CAR-T cell therapy, chimeric antigen receptor NK (CAR-NK) cells focus on natural killer cells, another protagonist in the human immune system, which play an important role in innate and adaptive immunity. Like γδT cells, NK cells take effect without the aid of MHC and are at low risk of GVHD. The activity of NK cells is coregulated by inhibition signals and activation signals. Most of the MHC-I molecules are inhibitory for NK cells and deregulated on tumor cells (73). With these superiorities, NK cells are the rising star of tumor immunotherapy. CAR-NK cells preserve natural killing functions independent of CAR, such as antibody-dependent cell cytotoxicity (ADCC) and cytolysis by secreting granzyme and perforin (74). In addition to PBMC, NK92 cell line, umbilical blood (UCB), CD34 hematopoietic progenitor cells (HPCs), and induced pluripotent stem cells (iPSCs) can also substitute or transform into NK cells. The ongoing clinical trials of CAR-NK cells are mainly based on NK92 cell lines and PBMC (75). Despite limitations such as the tumorigenic risk of the NK92 cell line and the short duration of the CAR-NK cells *in vivo* (73), CAR NK-cell therapy remains a promising direction as off-the-shelf cellular immunotherapy.

Hundreds of preclinical and clinical trials of CAR-NK cell therapy have been launched, with almost evenly splitting between solid tumors and hematological malignancies. In a clinical trial (NCT03056339) in CD19-positive lymphoid tumors, NK cells were transduced to express genes encoding anti-CD19 CAR, interleukin-15, and inducible caspase 9 as a safety switch. Of the 11 treated patients, 8 (73%) had a response, and 7 (64%) had a complete remission. Regarding safety, no cases of CRS or neurotoxicity were observed, neither any obvious increase in inflammatory cytokines nor GVHD with this HLA-mismatched CAR-NK product (nine partial matching at four of six HLA molecules and two non-HLA matched) (76). This preliminary study proves the safety advantages of CAR-NK cells in universal cell therapy.

### Induced Pluripotent Stem Cell

Induced pluripotent stem cell (iPSC) is a hotspot of research with unlimited capability to self-renew and differentiate into terminal cells, including T and NK cells with demonstrable antitumor activity. Besides, piles of homogeneous therapeutic cells from iPSC can be prefabricated, inspected, and banked across MHC barriers (77, 78). FT819, an iPSC-derived UCAR-T cell product expressing anti-CD19 CAR and antibody-engaging CD16 Fc receptor and TCR knockout, has shown the efficiency of controlling leukemia progression *in vitro* and *in vivo* in a mouse model, without alloreactivity (79). Maria Themeli et al. reported that iPSC-derived CAR-modifying T cells that resemble the phenotype of congenital γδT cells could effectively inhibit tumor growth in xenotransplantation models (80). Similarly, iPSC-derived CAR-NK cells demonstrated significant tumor inhibition and prolonged survival in the ovarian cancer xenograft model (15, 81). Nevertheless, the immortalization of iPSC also has both risks and opportunities, as the tumorigenic potential of undifferentiated iPSC has not been ruled out yet (48).

### Macrophage

Extracellular matrix (ECM) is very important for the development of malignant solid tumors and can act as a physical obstacle to various anticancer treatments, including CAR-T cells. Innate immune cells with phagocytosis activity, such as macrophages, can secrete matrix metalloproteinases (MMPs) to degrade almost all ECM components and penetrate tumors (82). Gene engineering with CARs imparted macrophages a sustained proinflammatory phenotype (M1) and antigen-specific phagocytosis (83). Recently, in two xenograft mouse models, CAR macrophages (CAR-M) targeting the solid tumor antigens mesothelin or HER2 decreased tumor burden and prolonged overall survival, which preliminarily proved its feasibility in solid tumors (84).

## Conclusions and Prospects

The achievements of CAR-T cell therapy in hematological malignancies have established cellular immunotherapy as a new pillar of antitumor therapy, but a series of limitations, such as high cost, low accessibility, and uncontrolled quality, have restricted its further dissemination and application. UCAR-T cell therapy is a comprehensive upgrade based on the original CAR-T cell therapy, which can remarkably improve accessibility and applicability. The gallop of gene-editing technologies and more plentiful cell sources have given it wings to reality. Many scientific and medical institutions and biotech companies have made initial successful attempts, although the persistence of UCAR-T cells is not as good as that of autologous CAR-T cells so far. In conclusion, the ultimate goal of UCAR-T cell therapy is to develop a conventional, living drug, just like blood transfusion, to provide a powerful booster for convenient, effective, and economical antitumor therapy. Current advances demonstrate that it is not a distant dream.

## Author Contributions

HL wrote the manuscript and was the primary author. LZ and WM made substantial contributions to designing and revising the article. All authors contributed to the article and approved the submitted version.

## Funding

This project was supported by the funding from the National Natural Science Foundation of China (No. 81900187).

## Conflict of Interest

The authors declare that the research was conducted in the absence of any commercial or financial relationships that could be construed as a potential conflict of interest.

## Publisher’s Note

All claims expressed in this article are solely those of the authors and do not necessarily represent those of their affiliated organizations, or those of the publisher, the editors and the reviewers. Any product that may be evaluated in this article, or claim that may be made by its manufacturer, is not guaranteed or endorsed by the publisher.
